# Unveiling the Biodiversity and Conservation Significance of Medog: A Camera-Trapping Survey on Mammals in the Southeastern Tibetan Mountains

**DOI:** 10.3390/ani14152188

**Published:** 2024-07-27

**Authors:** Qianqian Wang, Biao Yang, Ruifeng Zhu, Xin Wang, Shilin Li, Li Zhang

**Affiliations:** 1 Key Laboratory of Biodiversity and Ecological Engineering, Ministry of Education, College of Life Sciences, Beijing Normal University, Beijing 100875, China; qianqianwang212@163.com (Q.W.); asterzhang@bnu.edu.cn (L.Z.); 2 College of Life Sciences, China West Normal University, Nanchong 637001, China; 3 Society of Entrepreneurs and Ecology (SEE) Foundation, Beijing 100020, China; 4 Sichuan Zoological Society, Chengdu 610065, China; ruifengzhu66@gmail.com (R.Z.); 18060907642@163.com (X.W.); colinlee1994@hotmail.com (S.L.)

**Keywords:** camera trapping, Endangered species, mammals, Medog, occurrence, southeastern Tibetan mountains

## Abstract

**Simple Summary:**

The Medog region in southeastern Tibet harbors diverse wildlife, yet our understanding of these mammals’ species diversity, distribution, and conservation status remains inadequate. To address this, a camera-trapping survey was conducted in Gedang, Medog, spanning from April 2023 to May 2024. The study revealed 25 mammalian species across five orders and 14 families, including Endangered and Vulnerable species. Notably, the Gongshan muntjac’s underrepresentation in the IUCN was addressed, and new insights into the coexistence of Himalayan red pandas and Chinese red pandas were documented. The White-cheeked macaque, Gongshan muntjac, and Himalayan serow were frequently detected with high RAI. However, the high detection of domestic dogs, livestock, and human disturbances may pose threats to wildlife. Conservation efforts are crucial to protect these mammals and maintain ecological balance in the region.

**Abstract:**

The Medog in southeastern Tibet is home to a diverse range of wild animals. However, research on these mammals’ species directories, distribution, and conservation status remains insufficient, despite their crucial role in maintaining ecological balance. The study carried out a camera-trapping survey to assess mammal biodiversity and the significance of mammal protection in their natural habitats in Gedang, Medog. Future directions and application prospects of the study for wildlife conservation in the southeastern Tibetan mountains were also discussed. The survey, spanning from April 2023 to May 2024, with 19,754 camera trap days, revealed 25 mammalian species across five orders and 14 families. Among these, four classified as Endangered, five as Vulnerable, two as Near Threatened on the IUCN Red List, nine were categorized as Critically Endangered or Endangered on the Red List of China’s Vertebrates, and seven were China’s national first-class key protected wildlife. The order Carnivora exhibited the highest diversity, comprising 12 species. Furthermore, the study filled the knowledge gap regarding the underrepresentation of Gongshan muntjac *Muntiacus gongshanensis* in IUCN and provided new insights into the recorded coexistence of the Himalayan red panda *Ailurus fulgens* and Chinese red panda *Ailurus styani* along the Yarlung Zangbo River for the first time, and also documented new upper elevation limits for four large to medium-sized species. Regarding the relative abundance indices (RAI) captured by camera traps, the most prevalent species identified was the White-cheeked macaque *Macaca leucogenys*, followed by the Gongshan muntjac and Himalayan serow *Capricornis thar*. The monitoring also captured a number of domestic dogs and livestock, as well as human disturbances. These findings underscore the importance of conserving these mammals and emphasize the need for conservation efforts to protect their habitats and reduce human activities that threaten their survival, thereby maintaining the ecological balance of the region. Additionally, the research highlighted Gedang’s significance to global conservation efforts for mammalian diversity, providing essential data for effective wildlife conservation strategies.

## 1. Introduction

Studies have shown that the ongoing sixth mass extinction is accelerating, even worsening, with more than 500 land animal species expected to go extinct in the next 20 years, a rate that matches the dinosaur extinction 66 million years ago [1,2]. However, unlike previous mass extinctions, human activities are responsible for almost all the unprecedented biodiversity loss we see today [3]. There is no doubt that this mass extinction of thousands of animal and plant species has profound consequences for how ecosystems function and what they offer humanity, such as food, water, and disease control. Consequently, urgent actions are imperative for protecting and restoring the living world to prevent the collapse of civilization.

Fricke et al. [4] used deep learning models to simulate changes in global terrestrial mammal food webs over the past 130,000 years and showed that human-induced extinction and range loss have led to a 53% decline in network degree, resulting in a collapse of network connectivity, complexity, and stability. Nonetheless, mammals are a vital component of biodiversity, providing various ecosystem services and functions such as seed dispersal, pollination, predation, and nutrient cycling, owing to their life history and reproductive strategies [5]. Yet, they are also among the most threatened groups of animals, facing habitat loss, overexploitation, climate change, invasive species, etc. [6]. As research findings have shown, habitat destruction and overexploitation caused widespread declines in all aspects of mammalian diversity [7]. Therefore, understanding the biodiversity, distribution, abundance, and conservation status of mammals is crucial for their effective management and protection. One of the challenges for mammal research and conservation is the difficulty of detecting and monitoring them, especially in remote and complex habitats, such as tropical forests.

Located in southeastern Tibet and the lower reaches of the Yarlung Zangbo River, Motuo County (Medog) stands as one of the most remote and inaccessible areas in China, with a complex terrain and rich biodiversity, earning it the nickname “Low Valley of the Roof of the World”. Much of the Tibetan mountains remain poorly studied, with scarce records due to the geographically remote location, extreme climate, and hazardous geographical conditions [8]. Until October 2013, Medog had no roads connecting it to the outside world, contributing to its status as one of the least explored regions in China. Despite its isolation, reports indicate a high biodiversity with over 3000 species of higher plants, and many mammal species that are documented to be rare and endangered, such as Bengal tiger *Panthera tigris tigris*, Clouded leopard *Neofelis nebulosi*, Red goral *Naemorhedus baileyi*, and White-cheeked macaque *Macaca leucogenys* [9,10,11,12]. However, the exact extent of species diversity and conservation status, or whether these mammals face human-induced threats in Medog remains largely unknown due to the lack of baseline data necessary for systematic monitoring, surveys, and management, and there is no systematic research or reliable estimates of their density and abundance available.

Camera-trapping technology, a method that uses remotely triggered cameras to capture images of animals in their natural habitats, is an effective tool for studying wildlife populations and habitats [13]. This technique offers crucial information about mammalian diversity, distribution, occupancy, behavior, and activity patterns, especially useful for studying elusive and nocturnal species. Data from a camera trap can help to estimate population sizes, monitor habitat use, and study behavior patterns [14,15,16,17,18]. However, the use of it in the southeastern Tibetan mountains (STM) remains challenging due to the hazardous and mysterious nature of the environment, as well as restricted accessibility of the border regions [19]. In recent years, escalating anthropogenic disturbances, including local road construction, tourism development, and even poaching activities, may have exacerbated habitat loss and fragmentation for local wildlife, posing a potential threat to certain species’ survival [8,20]. Therefore, identifying mammal species in Medog and implementing protective measures are essential for preserving the ecological integrity and cultural heritage of the region.

In this study, we concentrated on researching the diversity and distribution status of mammal species in Gedang, Medog of Tibet Autonomous Region, China. We conducted a camera-trapping survey and a statistical analysis to evaluate the importance of their conservation. Thus, our objectives included compiling an inventory of species and providing an initial assessment of their relative abundance, to understand the potential conservation importance of the STM. Additionally, we assessed the preliminary threats and challenges to mammal conservation in the region and proposed recommendations for future actions.

## 2. Materials and Methods

### 2.1. Study Area

**Figure 1:**
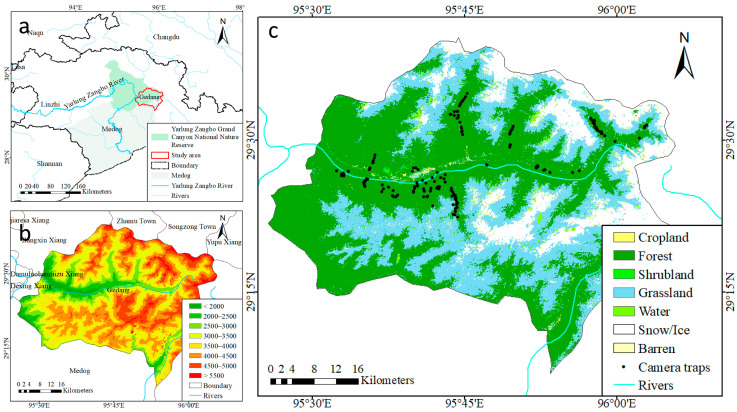
(**a**) Study area and adjacent regions of Gedang, Medog in Tibet Autonomous Region, China. (**b**) The Digital Elevation Model (DEM) derived from 1:1 million data, provided by the National Catalogue Service for Geographic Information (https://www.webmap.cn/main.do?method=index, accessed on 10 March 2024), and (**c**) the land use type using the 30 m annual land cover dataset from the Geospatial Data Cloud (https://www.gscloud.cn/search, accessed on 12 March 2024). The inset black dots represent the locations of camera traps.

Gedang, a township in Medog within the Tibet Autonomous Region, China, is situated within the Yarlung Zangbo Grand Canyon National Nature Reserve. This territory is a multi-ethnic gathering sparsely populated place, where the main ethnic groups include Tibetan, Monba, Lhoba, and Han. It is situated in the lower reaches of the Yarlung Zangbo River, on the southern slope of the Himalayas, and has an elevation range of 1327–6315 m (Figure 1a), with snow and ice covering the mountain peaks above 4000 m elevation throughout the year (Figure 1b,c). It is one of the lowest and warmest areas on the Tibetan Plateau, known for its unique climate and vegetation. The area experiences abundant rainfall, with the annual precipitation concentrated between June and August. Gedang covers a total area of 2917 km^2^, 83.66% of which is covered by vegetation, while the remaining area mainly consists of snow-capped mountains (Figure 1c). Its unique ecological environment supports a variety of vegetation types, mainly comprising coniferous forests on mountains in subtropical and tropical zones, subtropical evergreen broad-leaved forests, mixed coniferous and broad-leaved forests, shrublands, and alpine vegetation.

### 2.2. Camera Trapping

**Figure 2:**
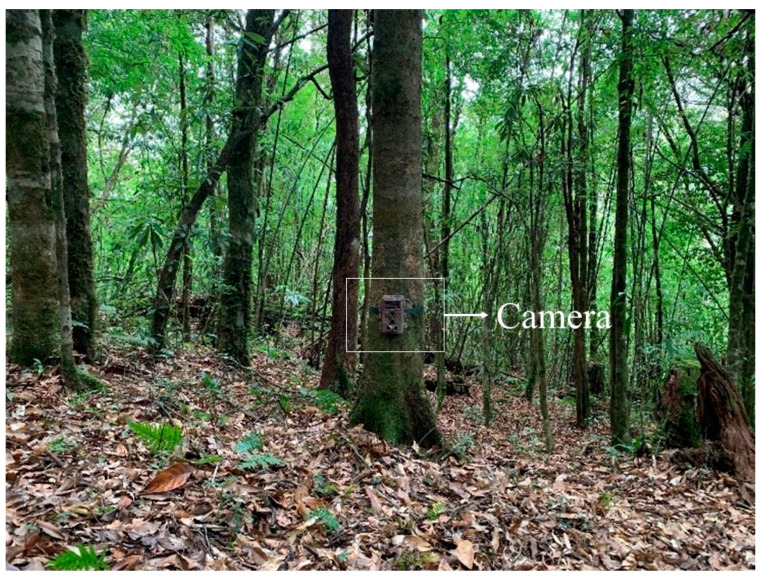
Schematic diagram of infrared camera installation.

Camera-trap surveys were conducted from April 2023 to May 2024 to monitor mammalian species in their natural habitats using 110 cameras (MT 660 series, Shenzhen Zhongye Yunhu Electronic Equipment Co., Ltd., Shenzhen, China). Each camera was configured to operate continuously for 24 h. Each event was registered by 3 photographs followed by a 20 s video, with a 30 s interval before the next trigger. Infrared cameras were mounted 0.4–0.6 m above the ground level and the camera lenses were adjusted to be parallel to the ground, considering the terrain (Figure 2). Camera traps were strategically positioned in areas of high wildlife activity, animal trails, ridge tops, and waterholes, at elevations from 1929 to 3654 m. The location of each camera trap station was recorded using GPS, and the mean distance between neighboring cameras was about 1 km. These camera traps were randomly distributed in broadleaf forests, conifer forests, shrublands, and alpine vegetation.

### 2.3. Data Analysis

The method of data processing used involved identifying mammalian species in the images, analyzing the data to estimate the frequency of animal occurrence, and combining a geographic information system (GIS) to analyze the distribution of these species. Large and medium-sized mammals were defined as species with a mean body mass greater than 0.5 kg [21]. The images captured by the camera trap were organized and managed using Bio-Photo (V2.1) software, and the identification of mammalian species in the images followed the criteria of Liu et al. [22]; Smith and Xie [23]; Wei et al. [24]; and Jiang et al. [25,26].

The conservation status of the species was assessed at both global and national levels according to the threat categories assigned in the IUCN Red List [27] and the Red List of China’s Vertebrates [28], respectively. Elevation ranges of the detected species with those previously found were compared. The survey effort was quantified by the total number of camera-days, where each 24 h continuous operation of an infrared camera in the field counts as one camera-day. A single capture event was defined as any sequence for a given species taken more than 30 min after the previous sequence of that species at the same location [29,30]. Additionally, we tallied the total number of independent capture events for each species and determined the percentage of camera traps (PC) that recorded each species [30,31]. The relative abundance indices (RAI) were also calculated according to the methods suggested by Carbone et al. [32] and O’Brien et al. [29], as a species quantitative index to assess the abundance of species within the territory [33,34,35]. The PC and RAI were calculated using these formulae:

$$PC=\left(\frac{S}{T}\right)\times 100\%;\;RAI=\frac{A_{i}}{N}\times 1000$$

where S is the number of camera traps that recorded the species; T represents the total number of camera traps; $A_{i}$ represents the number of independent mammal photographs of the i-th species; and N is the total number of camera-days [35,36].

## 3. Results

### 3.1. Mammalian Diversity in Gedang, Medog

**Figure 3:**
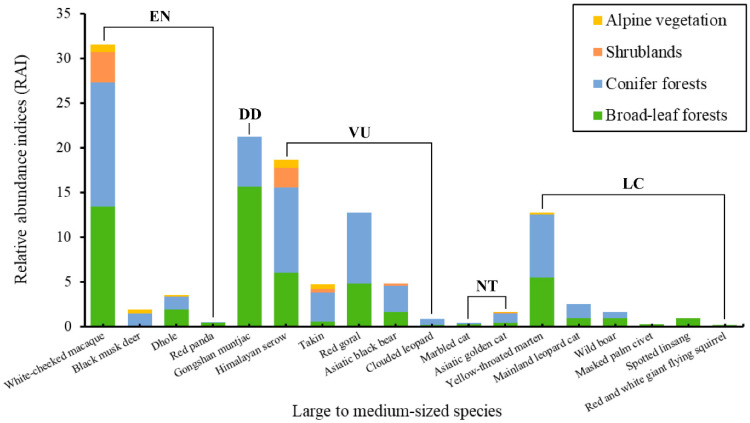
The relative abundance indices (RAI) for large to medium-sized species are depicted, with different colors in the columns representing the percentage of traps recording species across various habitat types. Uppercase letters on the line correspond to the IUCN Red List conservation status of species.

**Figure 4:**
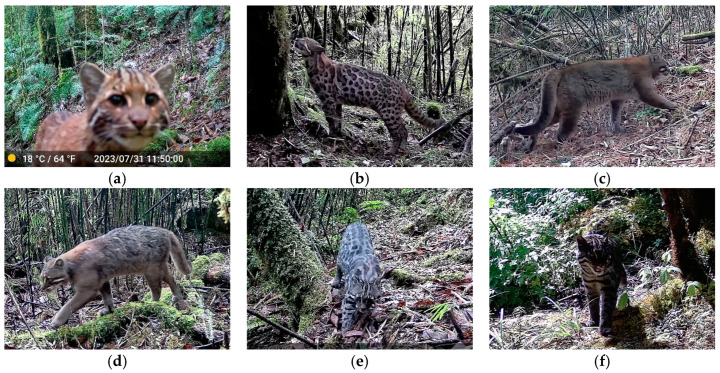
Diverse color types of the Asiatic golden cat *Catopuma temminckii* photographed by infrared camera traps include: (**a**) common golden; (**b**) golden with spotted; (**c**) dark cinnamon; (**d**) gradient ramp; (**e**) gray with spotted; (**f**) melanistic.

Among the 110 camera traps, 5 failed to generate usable data due to camera malfunctions or loss. Over 19,754 camera-days, a total of 21,973 mammal photos and videos were captured, with 3894 independent detections, of which 943 were rodent images of unspecified species, and 197 were people, livestock, and domestic dogs. In total, 25 species of mammals belonging to five orders and 14 families were identified, with 18 of them in the category of large and medium-sized mammals (Table 1; Figure 3). The order Carnivora, with its 12 species including four feline species, exhibited the highest diversity, particularly in the variety of body colors and patterns seen in the Asiatic golden cat *Catopuma temminckii* (Figure 4; Figure S1), while the order Cetartiodactyla followed with six species. Of these detected species, four were categorized as Endangered, five as Vulnerable, two as Near Threatened, and one as Data Deficient on the IUCN Red List. According to the Red List of China’s Vertebrates, six were categorized as Critically Endangered, three as Endangered, five as Vulnerable, and five as Near Threatened. Additionally, seven species were designated as China’s national first-class key protected wildlife (Table 1; Figure S1).

**Figure 5:**
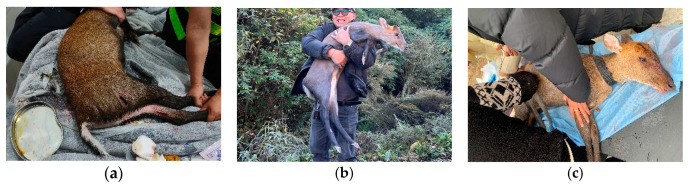
Samples of the Gongshan muntjac *Muntiacus gongshanensis* that were injured and received treatment during the research period. (**a**) On 18 May 2023, a rescue attempt for a bitten Gongshan muntjac failed; (**b**) On 31 December 2023, a Gongshan muntjac that was trapped at the base of the mountain was rescued and released; (**c**) On 5 February 2024, a Gongshan muntjac with a leg injury was successfully rescued.

**Figure 6:**
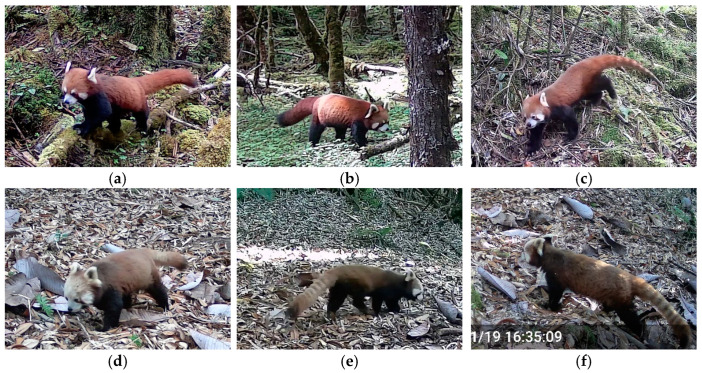
Infrared camera-trap images of the phenotypic differences between (**a**–**c**) the Chinese red panda *Ailurus styani* and (**d**–**f**) the Himalayan red panda *Ailurus fulgens*.

Notably, we discovered three species that are rare in China and even the world: Gongshan muntjac *Muntiacus gongshanensis*, Himalayan serow *Capricornis thar*, and Himalayan red panda *Ailurus fulgens*. The Gongshan muntjac *Muntiacus gongshanensis* is of particular interest, as data on this species is limited in the IUCN database (Table 1). Despite this, this species was frequently captured on our cameras and was actively targeted in our rescue efforts (Figure 5). Remarkably, the study area is inhabited by both the Himalayan red panda *Ailurus fulgens* and the Chinese red panda *Ailurus styani*. Although their distinction as separate species has been recently recognized, due to the IUCN’s lack of distinction, our study still considers them as ecotypes or subspecies of the Red panda (Table 1). In most (75%) capture events, they could be identified by facial features, even without distinctive white or dark tail rings. But in some cases, distinguishing between them species based on coloration with clear tail visibility alone was not feasible (Figure 6).

### 3.2. Mammalian Distribution in Gedang, Medog

From the camera-trap data on species distribution, the highest mammal abundance was observed in the broad-leaved forest, followed by the coniferous forest, with the lowest recorded in the alpine vegetation. Except unidentified rat, five species, namely the White-cheeked macaque *Macaca leucogenys*, Himalayan serow *Capricornis thar*, Takin *Budorcas taxicolor*, Asiatic golden cat *Catopuma temminckii*, and Siberian weasel *Mustela sibirica* were found across all the habitat of vegetation types at various elevations. By contrast, 19 other species only occurred in some specific habitats. These five species include the Himalayan red panda *Ailurus fulgens*, Masked palm civet *Paguma larvata*, Spotted linsang *Prionodon pardicolor*, Yellow-bellied weasel *Mustela kathiah*, and Red and white giant flying squirrel *Petaurista alborufus* were recorded only in broad-leaf forest habitat. The species with the widest distribution in terms of the elevational range was the White-cheeked macaque *Macaca leucogenys*, and the Yellow-bellied weasel *Mustela kathiah* was the most restricted species, recorded only once at an elevation of 2608 m. Moreover, new upper elevation limits were recorded for four large to medium-sized species: the White-cheeked macaque *Macaca leucogenys* at an elevation of 3502 m, the Gongshan muntjac *Muntiacus gongshanensis* at 3028 m, the Himalayan serow *Capricornis thar* at 3654 m, and the Marbled cat *Pardofelis marmorata* at 2900 m (Table 1).

### 3.3. Mammalian Relative Abundance Indices (RAI) in Gedang, Medog

The most prevalent species identified via camera traps, as indicated by the total number of independent capture events and the relative abundance within the entire monitoring area, was the White-cheeked macaque *Macaca leucogenys*, which belongs to Primates. In terms of RAI captured by camera traps, the second highest was the Gongshan muntjac *Muntiacus gongshanensis*, with an RAI of 21.26, followed by the Himalayan serow *Capricornis thar* with an RAI of 18.68, and the Red goral *Naemorhedus baileyi* with an RAI of 12.71. Conversely, the species with the lowest relative abundance that was recorded with no more than five independent detections, were the Masked palm civet *Paguma larvata*, Yellow-bellied weasel *Mustela kathiah*, Chinese red panda *Ailurus styani*, Masked palm civet *Paguma larvata*, Yellow-bellied weasel *Mustela kathiah* and the flying squirrel, each having an RAI value of less than 0.30. Additionally, the monitoring also captured a significant number of domestic dogs and livestock, as well as human disturbances such as tree felling, wood gathering, and medicinal herb collection, even hunting, all of which had a combined RAI of 9.97 (Figure S1). These data not only indicates the presence and prevalence of wildlife species but also highlights the impact of anthropogenic activities on the studied ecosystem.

## 4. Discussion

### 4.1. Diversity and Conservation Significance of Mammals in Gedang, Medog

This study used a camera-traps survey in conjunction with field observations and identified 25 mammal species across five orders and 14 families within the Gedang area, including several rare and Endangered species such as the Clouded leopard *Neofelis nebulosi*, Black musk deer *Moschus fuscus*, and Dhole *Cuon alpinus*. As a hotspot of biodiversity and endemism in China, the most diverse order of Gedang was Carnivora, which comprised 12 species, while Cetartiodactyla was represented by 6 species. Among these detected species, four were categorized as Endangered on the IUCN Red List, nine were marked as Critically Endangered or Endangered on the Red List of China’s Vertebrates, and seven were China’s national first-class key protected wildlife. The species’ distribution varied in terms of elevational range and habitat types. The study also recorded new maximum elevation limits for the White-cheeked macaque *Macaca leucogenys*, Gongshan muntjac *Muntiacus gongshanensis*, Marbled cat *Pardofelis marmorata*, and Himalayan serow *Capricornis thar*. The findings certainly highlighted the diverse and intact mammal community in Gedang and demonstrated that the STM holds significant potential for mammal conservation. However, despite its ecological significance, the conservation status of mammals in this region remains not well-known; hence, there is an urgent need to assess the population size, distribution, and threats faced by these species, particularly the endemic and threatened ones, and update their conservation status accordingly.

To our knowledge, the phylogenetic analysis supports the recognition of Gongshan muntjac as a distinct species within the genus *Muntiacus*, and Tibetan Yarlung Zangbo Grand Canyon National Nature Reserve in the Eastern Himalayas houses a rich diversity of muntjac species [40,41]. This research addresses the knowledge gap regarding the Gongshan muntjac and sheds light on its conservation status and habitat requirements, which have been understudied by the IUCN Red List due to scarce data. Additionally, our research revealed a wide coexistence of the jackal pack with other large or medium carnivore species, while the Dhole may be functionally extinct within the giant panda’s distribution range and other regions in China [42]. Furthermore, the mammal captured with the highest RAI was the White-cheeked macaque. Gedang is recognized as its type locality, as it was first discovered and classified there in 2015, and it also represents the second new primate species discovered in China since 2013 [43]. Primates are one of the most species-rich groups of mammals, surpassed only by the Chiroptera and Rodentia [44]. However, despite the prevalence of the White-cheeked macaque, our camera traps did not capture images of the Tibetan macaque *Macaca thibetana*, Rhesus monkey *Macaca mulatta*, Assamese macaque *Macaca assamensis*, Capped langur *Trachypithecus shortridgei*, or other potentially distributed primates reported to exist in Tibet [45,46,47]. Geographical barriers like mountains and rivers might have prevented the existence of these primates from being detected by our camera traps.

The Tibetan mountains provide a rich habitat for many carnivorous animals, especially notable for felid species [8,9,37], which was also observed in the current study. Here, four distinct species were found: Marbled cat, Clouded leopard, Mainland leopard cat *Prionailurus bengalensis*, and Asiatic golden cat *Catopuma temminckii*. Among these, the Mainland leopard cat exhibited the highest relative abundance, while the Marbled cat was less common, and the body colors and patterns of the Asiatic golden cat were more diverse and varied than those found in previous studies [48]. Flanked by China and India, the Medog has been repeatedly reported to harbor the transboundary large carnivore, the Bengal tiger *Panthera tigris tigris* [11,49]. In Nepal, successful conservation efforts have led to a significant recovery in the tiger population [50,51]; meanwhile, leopards in Nepal are more prevalent in areas with protected status, sufficient prey like wild boar, and less human activity [52]. However, the absence of these large fields in our research indicates a need for balanced conservation strategies or innovative solutions to increase their populations.

Notably, our research confirms that both the Himalayan red panda *Ailurus fulgens* and the Chinese red panda *Ailurus styani* coexist in this area. Groves [53] first proposed and recognized two distinct species based on morphological differences. However, the classification into separate species or subspecies has been controversial due to a lack of genetic evidence and uncertainty about their geographic distribution boundaries [53,54,55,56]. It was not until Hu et al. [57] provided comprehensive genetic evidence of their divergence and showed that the morphological differences used to distinguish them aligned with genetic variations and geographic distribution. Genomic evidence revealed that the red pandas living in southeastern Tibetan mountains and northern Myanmar belong to the Chinese red panda, while those inhabiting southern Tibetan mountains are the Himalayan red panda, along with Nepalese individuals, and the Yarlung Zangbo River is most likely the largest geographic barrier to their distribution. Our research provides the first confirmation that both of them coexist at a bend in the lower Yarlung Zangbo River. The Chinese red panda typically has redder facial fur with less white, forming a ‘visible mask’, while the Himalayan red panda shares similar morphology but has more white in its facial fur [57]. Interestingly, the Himalayan red pandas we photographed exhibited an overall color slightly different from previous reports, leaning toward a more brownish hue (Figure 6). However, their small population size with low RAI value underscores the critical need to maintain and increase genetic diversity for its long-term persistence. Specifically, the Himalayan red panda population spans southern Tibet of China, Nepal, India, and Bhutan, necessitating urgent transboundary international cooperation to protect this declining species. The pictorial distribution of these species in the map of Nepal also showed that the Asiatic black bear *Ursus thibetanus* has a much more extensive distribution than the red panda [58], which was highly consistent with our results, while studies on suitable habitat for the red panda in China are lacking. As a vegetarian within the carnivore order, similar to the Giant panda *Ailuropoda melanoleuca*, there remains much to discover about this species in the future.

### 4.2. Application Prospects of the Camera-Trapping Survey in the STM

A camera-trapping survey was utilized to document the mammal assemblage in Gedang, demonstrating the effectiveness of this technology in monitoring mammals and underscoring the importance of conservation in the area. Data from camera trapping can be processed using a statistical model to estimate the mammal density and determine the impact of protected areas on diversity [59,60]. Although camera trapping has been widely used for monitoring and studying terrestrial mammal diversity, distribution, and behavior, particularly for elusive and nocturnal species, its application for arboreal mammals or species with low migration and population density is still limited [31,34]. The survey has yet to detect certain species known to inhabit the Medog region, including the Bengal tiger, the Chinese serow *Capricornis milneedwardsii*, Northern red muntjac *Muntiacus vaginalis*, among others [12,37,49]. Bruce et al. [21] conducted a camera-trapping survey of large and medium-sized mammals in the Duga Wildlife Reserve in Cameroon and found distinct differences in mammal communities at two sites 32 km apart. Therefore, future camera-trapping efforts should be more extensive and systematic covering a wider elevational range and a variety of microhabitats, both on the ground and in the canopies, for monitoring mammal diversity and dynamics, thereby enhancing the likelihood of detecting unrecorded species.

Meanwhile, camera trapping is a powerful tool for monitoring elusive and enigmatic wildlife, detecting various threats like habitat loss, poaching, and human-wildlife conflict to native mammals, thereby helping to maintain ecosystem balance [33,60]. For instance, in Nepal, the distribution of the Red panda has been compromised by human-induced fragmentation and habitat loss, while key factors like proximity to paths, livestock and human densities, and temperature influence their habitat suitability. Concurrently, the population of the Asiatic black bear has dwindled due to deforestation and poaching pressures [58,61,62]. The survival and development of wild animals depend heavily on habitat suitability. As humans and wild animals’ habitats start to overlap, encounters with wild animals have increased [63]. Habitat loss due to human development and disturbances in forests may contribute to human–wildlife conflicts and declining wildlife populations [64]. A decline in species abundance can lead to inbreeding, population fragmentation, and a reduction in genetic variation due to habitat fragmentation, loss of connectivity, bottlenecks, or genetic drift [65]. Therefore, implementing effective conservation measures, including long-term monitoring, is essential to protect and restore mammal diversity and ecosystem services. This involves using camera trapping and other methods to collect and analyze data on population size, habitat distribution, and trends of mammal species in the STM, evaluating the effectiveness and impacts of conservation measures.

### 4.3. Future Conservation Directions

In the present study, people, livestock, and domestic dogs were also recorded frequently, suggesting that the fauna may be potentially threatened by human disturbance (Figure S1). In border areas where poaching is a significant threat to the remaining wild tigers and facilitates the illegal wildlife trade, Nepal’s national park rangers are equipped with anti-poaching skills and advanced camera systems to monitor tigers, their prey, and potential poaching activities in real-time [66]. To continuously increase the Bengal tiger populations in India and Nepal, conservation efforts have involved local communities, which has yielded remarkable results [51,67]. In the trans-Himalayan region of northern India, human disturbances, particularly tourism and waste management, have significantly affected the coexistence of the Snow leopard *Panthera uncia*, Red fox *Vulpes vulpes*, and Leopard cat *Prionailurus bengalensis*, intensifying resource competition [68,69]. Human activities associated with the discovery of wild animals in their natural habitats highlight the high biodiversity and endemism of the STM and underscore the urgent need for its conservation. Significantly, strict protection measures must be enforced to prevent illegal activities such as logging, mining, hunting, and trapping, thereby safeguarding core habitats for mammal species. Furthermore, promoting community-based conservation involves local people in the managing and monitoring of the nature reserve, and providing alternative livelihoods and incentives for them to conserve the natural resources and wildlife. Lastly, environmental education needs to be enhanced by raising public awareness and appreciation of the value and importance of mammal diversity and ecosystem services, and responsible and sustainable behaviors and practices need to be encouraged.

## 5. Conclusions

In conclusion, Gedang harbors a diverse and unique mammal assemblage, which includes diversiform Endangered and endemic species. This richness holds immense conservation value, not only for the region and the STM but also for the global ecosystem. The presence of human disturbance or other potential threats underscores the urgency for immediate habitat conservation actions and conflict mitigation strategies. The research provides essential data and lays the scientific groundwork for developing and implementing effective conservation measures for the mammal fauna of Gedang, supporting the importance of conservation efforts in Gedang and its surrounding areas. Additionally, the mammal conservation significance of Gedang will provide valuable insights for protecting mammal diversity and promoting effective wildlife conservation. We hope to achieve the vision of Gedang as a wilderness sanctuary and paradise for mammals and other wildlife, as well as a model for biodiversity conservation in China and worldwide.

## Figures and Tables

**Table 1:** Mammal species monitored in the Gedang, Medog of Tibet Autonomous Region, China, along with their conservation status on the IUCN Red List [27] and the Red List of China’s Vertebrates [26]. Elevational limits from literature prevail over IUCN specifications, and habitat types refer to the dominant vegetation where each species resides.

Species (by Order)		IUCN Red List Status ^a^	China’s Vertebrates Red List Status ^b^	China’s National Protected Category ^c^	Previously Known Elevational Limits (m) ^d^	Elevational Range Observed (m)	Habitat Types ^e^	% of Traps That Recorded the Species (PC)	Total No. of Independent Capture Events	No. of Independent Detection	Relative Abundance Index (RAI)
**Primates**											
Cercopithecidae											
White-cheeked macaque *Macaca leucogenys*		EN	CR	II	1395–2700	1929–3502	A, B, C, S	0.71	5228	623	31.54
**Cetartiodactyla**											
Cervidae											
Gongshan muntjac *Muntiacus gongshanensis*		DD	CR	II	900–3000	1929–3028	B, C	0.29	2337	420	21.26
Bovidae											
Himalayan serow *Capricornis thar*		VU	EN	I	200–3000	1929–3654	A, B, C, S	0.56	2847	369	18.68
Takin *Budorcas taxicolor*		VU	VU	I	1000–4000	2031–3502	A, B, C, S	0.24	1268	93	4.71
Red goral *Naemorhedus baileyi*		VU	EN	I	2000–4500	2337–3362	B, C	0.23	1274	251	12.71
Moschidae											
Black musk deer *Moschus fuscus*		EN	CR	I	2600–4214 ^f^	2995–3654	A, C	0.04	116	38	1.92
Suidae											
Wild boar *Sus scrofa*		LC	LC	/	0–4813 ^g^	2188–2797	B, C	0.11	240	31	1.57
**Carnivora**											
Ursidae											
Asiatic black bear *Ursus thibetanus*		VU	VU	II	0–4300	2013–3480	B, C, S	0.40	357	95	4.81
Felidae											
Marbled cat *Pardofelis marmorata*		NT	CR	II	0–2637 ^f^	2013–2900	B, C	0.03	32	8	0.40
Mainland leopard cat *Prionailurus bengalensis*		LC	VU	II	0–4474 ^h^	2031–2900	B, C	0.18	195	50	2.53
Asiatic golden cat *Catopuma temminckii*		NT	CR	I	0–4282 ^i^	2203–3502	A, B, C, S	0.19	117	32	1.62
Clouded leopard *Neofelis nebulosa*		VU	CR	I	0–3500	2423–3010	B, C	0.11	82	17	0.86
Canidae											
Dhole *Cuon alpinus*		EN	EN	I	0–5300	1942–3480	A, B, C	0.22	397	69	3.49
Ailuridae											
Red panda	Chinese red panda *Ailurus styani*	EN	VU	II	2500–4800	3028–3420	B, C	0.03	15	3	0.15
	Himalayan red panda *Ailurus fulgens*					2423–3068	B	0.06	33	7	0.35
Viverridae											
Masked palm civet *Paguma larvata*		LC	NT	II	20–2700	1993–2423	B	0.04	20	5	0.25
Spotted linsang *Prionodon pardicolor*		LC	VU	II	150–3308	2013–3068	B	0.09	65	18	0.91
Mustelidae											
Yellow-throated marten *Martes flavigula*		LC	NT	II	0–4510	1936–3480	A, B, C	0.48	955	252	12.76
Siberian weasel *Mustela sibirica*		LC	LC	/	0–4875	2031–3502	A, B, C, S	0.23	152	49	2.47
Yellow-bellied weasel *Mustela kathiah*		LC	NT	/	0–4000	2608	B	0.01	3	1	0.05
**Rodentia**											
Sciuridae											
Pallas’s squirrel *Callosciurus erythraeus*		LC	LC	/	200–3000	1942–3028	B, C	0.15	539	152	7.69
Orange bellied Himalayan squirrel *Dremomys lokriah*		LC	NT	/	1500–3400	2300–3028	B, C	0.18	456	141	7.14
Red and white giant flying squirrel *Petaurista alborufus*		LC	LC	/	800–3500	2053	B	0.01	12	3	0.15
Grey-headed flying squirrel *Petaurista caniceps*		LC	LC	/	/	2800–3068	B, C	0.03	19	5	0.25
Muridae											
Unidentified rat		/	/	/	/	1929–3502	A, B, C, S	0.44	3430	943	47.74
**Lagomorpha**											
Ochotonidae											
Forrest’s pika *Ochotona forresti*		LC	NT	/	2600–4400	2581–3589	C, S	0.05	137	22	1.11

^a,b^ EN, Endangered; VU, Vulnerable; NT, Near Threatened; LC, Least Concern; CR, Critically Endangered; DD, Data Deficient; ^c^ I, China national first-class key protected wildlife; II, China national second-class key protected wildlife; ^e^ A, Alpine vegetation; B, Broad-leaf forests; C, Conifer forests; S, Shrublands; ^d,f,g,h,i^ According to IUCN [27]; Shi et al. [37]; Xu et al. [36]; Thapa et al. [38]; and Dhendup et al. [39].

## Data Availability

The data presented in this study are available on request from the corresponding author.

## Supplementary Materials

The following supporting information can be downloaded at: https://www.mdpi.com/article/10.3390/ani14152188/s1, Figure S1. Infrared camera-trap images captured partial mammal species and livestock, domestic dogs, and human activity within Gedang, Medog of Tibet Autonomous Region, China; No. of independent detection; Total no. of independent capture events.
