# Supplementary material for: Unveiling the Biodiversity and Conservation Significance of Medog: A Camera-Trapping Survey on Mammals in the Southeastern Tibetan Mountains
Source: Animals (Basel). 2024 Jul 27;14(15):2188. doi: 10.3390/ani14152188 (PMC11311013; doi:10.3390/ani14152188)
Supplement: Supplementary file 1 [file animals-14-02188-s001.zip › Figure S1.pdf]

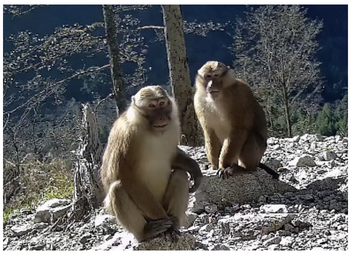

White-cheeked macaque

Macaca leucogenys

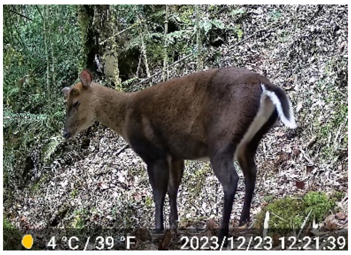

Gongshan muntjac

Muntiacus gongshanensis

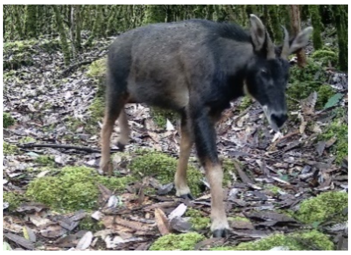

Himalayan serow

Capricornis thar

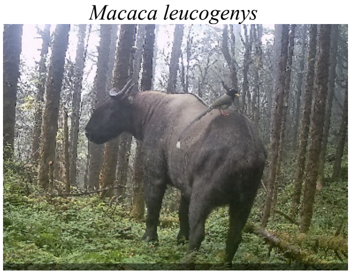

Takin (a)

Budorcas taxicolor

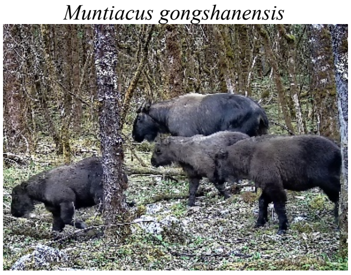

Takin (b)

Budorcas taxicolor

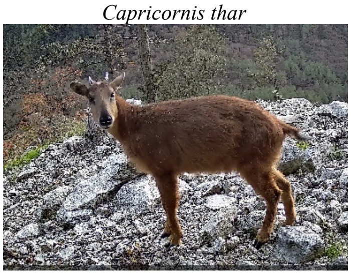

Red goral

Naemorhedus baileyi

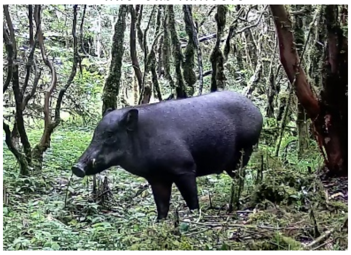

Wild boar

Sus scrofa

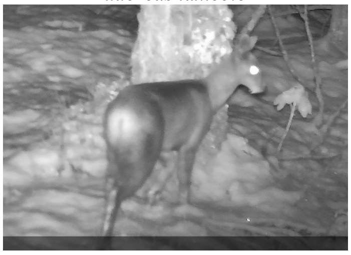

Asiatic black bear

Ursus thibetanus

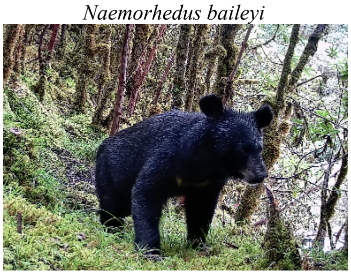

Asiatic black bear

Ursus thibetanus

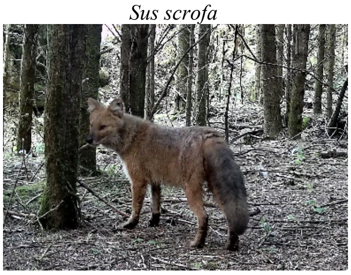

Dhole (a)

Cuon alpinus

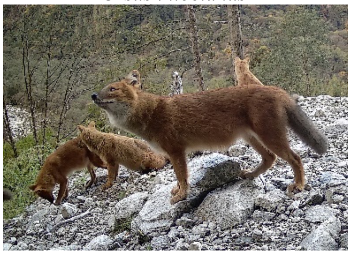

Dhole (b)

Cuon alpinus

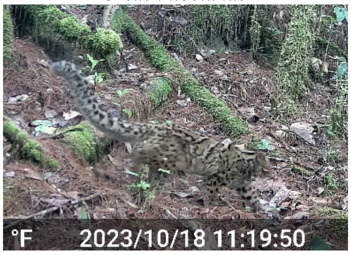

Marbled cat

Pardofelis marmorata

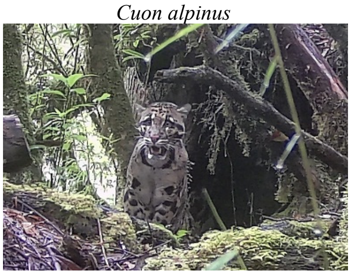

Clouded leopard (a)

Neofelis nebulosa

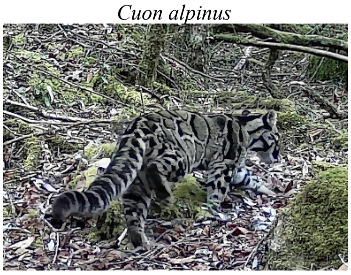

Clouded leopard (b)

Neofelis nebulosa

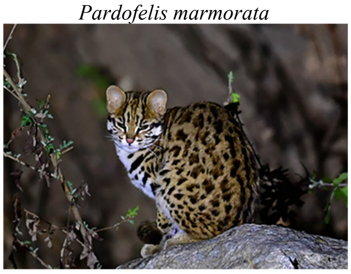

Mainland leopard cat (a)

Prionailurus bengalensis

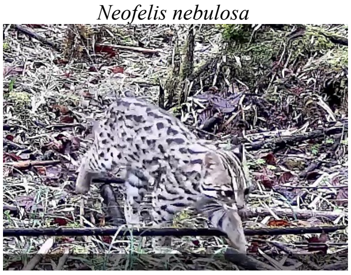

Mainland leopard cat (b)

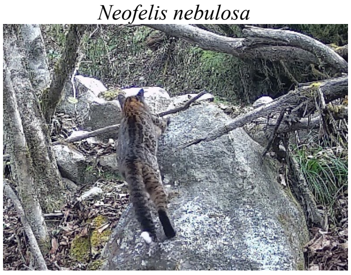

Asiatic golden cat (a)

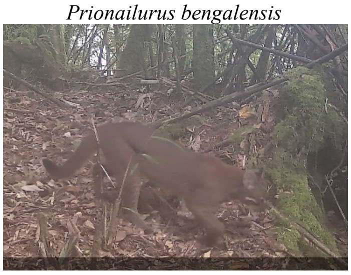

Asiatic golden cat (b)

Prionailurus bengalensis *Catopuma temminckii* *Catopuma temminckii*

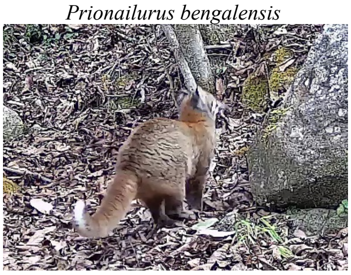
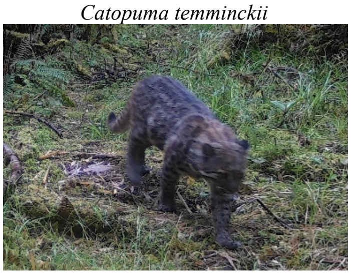
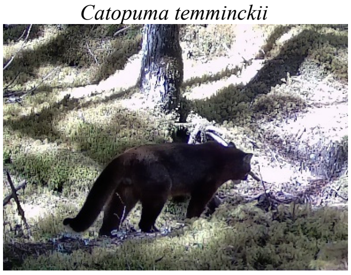

Asiatic golden cat (c) Asiatic golden cat (d) Asiatic golden cat (e)

Catopuma temminckii *Catopuma temminckii* *Catopuma temminckii*

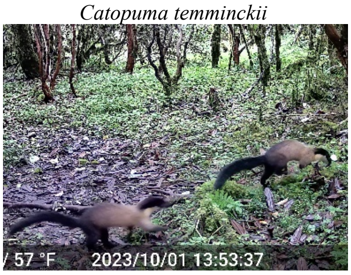

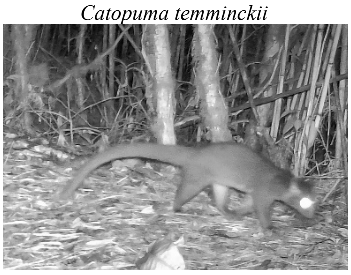
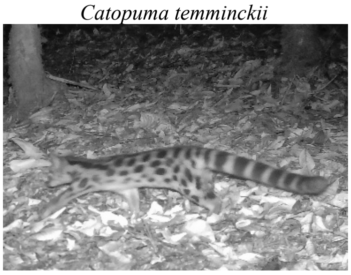

Yellow-throated marten Masked palm civet Spotted linsang

Martes flavigula *Paguma larvata* *Prionodon pardicolor*

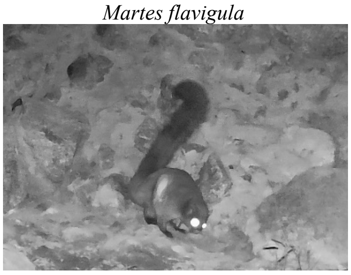
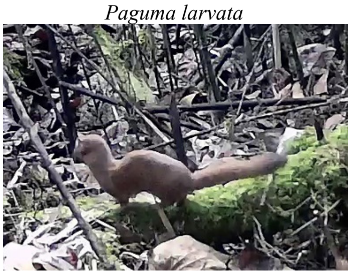
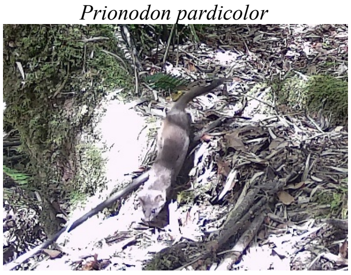

Red and white giant flying squirrel Siberian weasel Yellow-bellied weasel

Petaurista alborufus *Mustela sibirica* *Mustela kathiah*

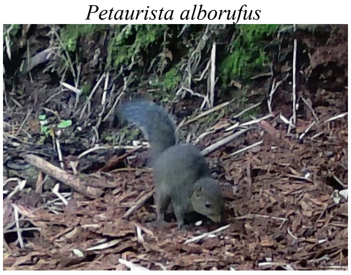
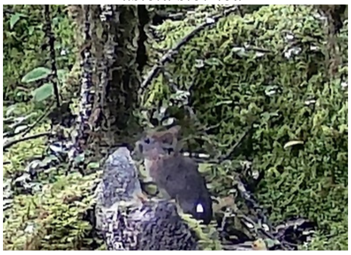
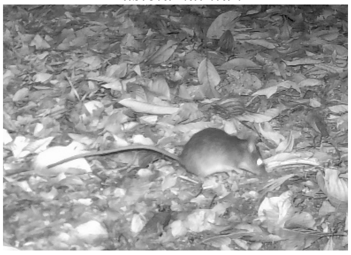

Orange bellied Himalayan squirrel Forrest's pika Unidentified rat

Dremomys lokriah *Ochotona forresti*

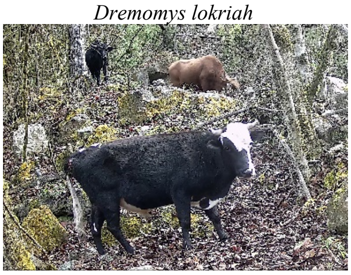
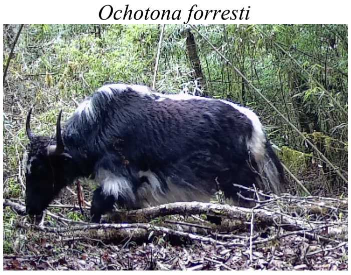
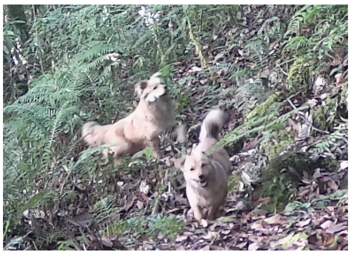

Livestock Yak Domestic dogs (a)

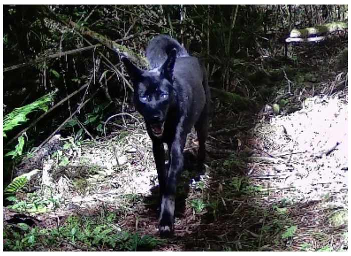
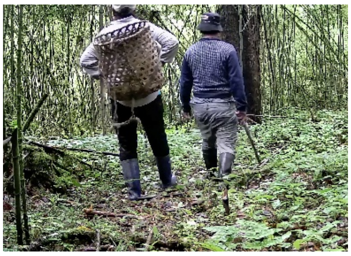
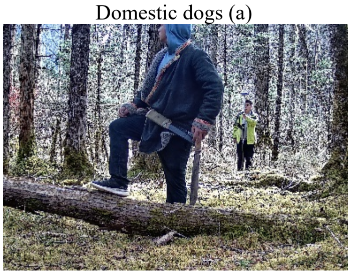

Domestic dogs (b) Human disturbin (a) Human disturbing (b)

Figure S1. Infrared camera-trap images captured partial mammal species and livestock, domestic dogs, and human activity within Gedang, Medog of Tibet Autonomous Region, China.
